# Breast awareness mobile apps for health education and promotion for breast cancer

**DOI:** 10.3389/fpubh.2022.951641

**Published:** 2022-10-05

**Authors:** Azlina Yusuf, Yulita Hanum P. Iskandar, Imi Sairi Ab Hadi, Arryana Nasution, Soon Lean Keng

**Affiliations:** ^1^School of Health Sciences, Universiti Sains Malaysia, Kubang Kerian, Malaysia; ^2^Graduate School of Business, Universiti Sains Malaysia, Penang, Malaysia; ^3^Department of Surgery, Hospital Raja Perempuan Zainab II, Kota Bharu, Malaysia

**Keywords:** mobile phone apps, breast cancer, early detection of breast cancer, breast self-examination, knowledge, awareness

## Abstract

**Background:**

Lack of knowledge, poor awareness, and attitude are barriers to breast cancer (BC) screening participation. The ubiquitous usage of mobile phones makes it a perfect platform for delivering interventions to increase knowledge and awareness in screening, a strategy for early identification of BC. However, although numerous applications for BC prevention are available on major mobile phone platforms, relatively few have been tested in scientific studies to determine their efficacy.

**Objective:**

This study aimed to assess the efficacy of BrAware Apps in increasing the knowledge of BC risk factors, awareness of warning signs and confidence in breast self-examination (BSE) among women in northeast peninsular Malaysia.

**Methods:**

A quasi-experimental pre and post-test research design were conducted with 41 women participants in Kelantan, Malaysia, before and after using the BrAware apps. Participants were given an online, adapted Breast Cancer Awareness Measure questionnaire. Post-test was 2 months after using the BrAware apps. Comparison using paired *T*-tests were conducted to evaluate the change in knowledge of risk factors, warning signs awareness and confidence level for BSE.

**Results:**

The mean age of women was 39.71(SD = 8.80). The participants' mean knowledge score of BC warning signs differs before using BrAware (mean 70.62, SD 11.74) and after using the BrAware app (mean 79.83, SD 10.15) at the <0.001 level of significance.

**Conclusions:**

The BrAware mobile app had a positive effect in increasing the women's knowledge of risk factors of BC, warning signs awareness and confidence level for BSE. It can be concluded that the mobile app may be an adjunct in educating women on BC.

## Introduction

Breast cancer (BC) is the most common cancer globally (1). It accounts for about 30% of female cancers and a mortality-to-incidence ratio of 15% as women's first oncological cause of death (2). Similarly, BC was the most often diagnosed malignancy among Malaysian women from 2007 to 2011, accounting for roughly 32.1% of all cases. According to the National Cancer Institute, the 5-years survival rate for BC in Malaysia is around 87.5% for stage I, 80.7% for stage II, 59.7% for stage III, and 23.3% for stage IV from 2007 to 2016 (3).

Screening for BC is available in Malaysia, and diagnosing the disease sooner and treating it in its early stages is feasible. Many measures have been launched in Malaysia to prevent BC mortality and morbidity by raising awareness through campaigns and screening programmes offered by government institutions. BC awareness month, often known as “Pink October,” has aided in raising awareness among women. However, the number of women who regularly get screened is far from satisfactory. A lack of knowledge of the various cancer screening methods, cultural attitudes, and a lack of encouragement by family members and doctors are the major reasons for the poor response to screening. Despite being an upper-middle-income nation with a robust healthcare system and effective socioeconomic initiatives, Malaysia's cancer survival rates are lower than the global average. The rate is attributable to several factors, including poor cancer awareness and screening rates, delays in seeking medical help, delays in detection and diagnosis, and insufficient access to high-quality care. These barriers are particularly obvious for people who live in rural areas because cancer centers are typically located near major cities (4). Therefore, Malaysia's awareness program for BC and health promotion and education needs to be reinforced.

As the use of mobile phones grows, so does the need for mobile phone apps. These applications might be used for various purposes, including social engagement, education, entertainment, and personal health. Mobile applications have positively impacted health-related behaviors and clinical health outcomes. Application users were more satisfied with using mobile health applications to manage their health than users of conventional care (5). A mobile app for the women population at risk in Malaysia was developed as a new tool for BC's health education and promotion. This study has shown its usability (6). However, we do not know whether this mobile app can improve users' knowledge, attitudes, and behavioral changes on BC. Therefore, this study aimed to assess the efficacy of the BrAware mobile app in improving the knowledge of risk factors of BC, warning signs awareness and confidence level for BSE among women aged 18 years and older who are the population at risk for the disease in northeast peninsular Malaysia.

## Methods

### Research design and study participants

A quasi-experimental pre and post-test research design were conducted among women in Kelantan, Malaysia, to measure the efficacy of a mobile application (BrAware) in increasing the knowledge of BC risk factors, awareness of warning signs, and confidence in breast self-examination (BSE). The state of Kelantan is largely rural, and its culture is quite distinct from other Malaysian states (7). The inclusion criteria were women aged 18 years and above who owned a smartphone and had never been diagnosed with BC. No age range was specified because the risk of BC increases with age. In addition, participants were excluded if their self-reported mobile app literacy was low and the phone did not run appropriately after downloading the mobile application. Low mobile app literacy is the inability to find, use, understand and evaluate the apps (8). A non-probability sampling method using social media such as Facebook and WhatsApp group were used to recruit public women living in Kelantan. The sampling technique was considered because it was more cost-effective and time-effective than probability sampling, and it was also impossible to do a probability sampling (9).

### Instrument

The questionnaire consisted of two sections: sociodemographic and BC awareness. Sociodemographic variables included age, occupation, monthly household income, ethnicity, race, highest education level, BC family history, trained BSE, and period to seek medical help if there was a change in the breast). The Breast Cancer Awareness Measure (B-CAM) and B-CAM-M (M for Malay language) were validated in the United Kingdom (10), and Malaysia (11) were adapted. The content and response format were modified to make it more culturally relevant for the local women. Of the seven domains of the B-CAM, three domains were included: Knowledge of risk factors, awareness of warning signs, and BSE. Nineteen items were adapted for BC awareness (9 items on knowledge of risk factors, eight items on awareness of warning signs and two items on confidence in BSE). A 5-point Likert scale was used: strongly agree, agree, neutral, disagree, and strongly disagree. The total score for each domain was summated into a percentage score. The mean percentage score of BC awareness before and after using the BrAware App was computed and compared. Findings showed that the BC awareness Cronbach alphas coefficient was 0.89, indicating good internal consistency (12). The instruments were pilot tested on ten women similar to the samples but not included in the final sample. No changes were made to the instruments.

### Procedure

Using the Malay language, data was collected online using the Google Forms platform between October 1, 2021, and December 1, 2021. This study applied various strategies to maintain social distance and observe the Movement Control Order. These include relying on the researchers' professional and personal networks to publicize and disseminate the advertisement *via* posters and social media like Facebook and WhatsApp and sharing the poster through email. An introduction statement and instructions for completing the pre and post-test online and a link for participants to download the BrAware App are included in the information. Participants were expected to become familiar with the BrAware App. The researchers would remind the subjects to complete a post-test 2 months after the pre-test date through Whats App text message or phone call. Instructions for completing the questionnaire online were also supplied by clicking the “Continue” button. Participants were given a choice to respond to the survey using the “Yes/No” option to confirm their willingness to participate. The participant was instructed to finish the online questionnaire after receiving confirmation to continue “Yes,” whilst “No” indicates a refusal to participate in the survey. To avoid duplicated or exaggerated data, participants were limited to one response. The survey took ~10–15 min to complete. For the post-test, the same instrument was employed. Therefore, the data set only included participants who completed both the pre-test and post-test.

### BrAware app

The mobile app, BrAware, from the abbreviation of Breast Awareness, was developed based on the Android platform by the researchers (6). The app was user-friendly, constructed with simple point form sentences, including a share button, infographic images or video, dual language capabilities (English and Malay language), a Google Map navigator and a reminder function. BrAware App content includes information about breast anatomy, BC, risk factors of BC, treatment modalities, BSE, screening examination, doctor examination, survival rate, finding support group, hotline number, screening reminders and myths and facts based on Malaysian cultural beliefs.

### Statistical analysis

Collected data were coded and analyzed using IBM Statistical Package for Social Sciences (SPSS) (version 26, IBM Corp., Armonk, NY). Descriptive data were used to describe the characteristics of sociodemographic variables. The test score is presented as means ± standard deviation (SD) and analyzed by a paired *t*-test. *P* < 0.05 is considered statistically significant.

## Results

A total of 41 women completed the online survey questionnaire. Sociodemographic characteristics are shown in Table 1. The mean age was 39.71 (SD = 8.80). The ethnic distribution of participants was 95.1% Malay while the remainder, 4.8%, was non-Malay. More than half (51.2%) of the participants obtain a secondary education. Most of the participants (78%) were married women. The mean household income of participants was 5,582.93 (SD = 8,158.21), and 9.8% had a BC family history, whilst 24.4% were not trained in BSE. The mean period to seek medical help if found a change in the breast was 6.29 (SD = 10.72).

**Table 1:** Socio-demographic characteristics of participants (*n* = 41).

**Variables**	**Mean (SD)**	***n***	**%**
**Age (years)**	39.71 (8.80)		
20–29		7	17.1
30–39		10	24.4
40–49		21	51.2
≥50		3	7.3
Range	22–63		
**Occupation**			
Housewife		7	17.1
Self-employed		5	12.2
Working (private)		13	31.7
Working (government)		16	39.0
**Monthly household income (MYR)**	5582.93 (8158.21)		
Range	0–50000		
Median	2800	24	58.5
≤ RM 4850.00		17	41.5
>RM 4850.00			
**Ethnicity**			
Malay		39	95.1
Non-malay		2	4.8
**Marital status**			
Single		2	4.9
Married		32	78.0
Widowed		7	17.1
**Highest education level**			
Postgraduate		6	14.6
Degree		10	24.4
Diploma		4	9.8
Secondary		21	51.2
**BC family history**			
Yes		4	9.8
No		37	90.2
**Trained BSE**			
Yes		31	75.6
No		10	24.4
**Period to seek medical help if found a change in breast (days)**			
Range	6.29 (10.72)		
Median	0–60		
Immediate	3	10	24.4
≤ 3 days		12	29.3
>3 days		19	46.3

MYR, The Malaysian Ringgit, the currency of Malaysia (MYR4.22 equal to 1 United State Dollar as of April 7th 2022).

Results of the paired *t*-test show that the mean knowledge score of BC warning signs differs before using BrAware (mean 70.62, SD 11.74) and after using BrAware (mean 79.83, SD 10.15) at the <0.001 level of significance. On average, knowledge of BC warning signs was about 9.21 points higher after using BrAware. However, for BSE knowledge, the mean score before and after using BrAware was increased to 9.75 (*p* = 0.007). In addition, the mean knowledge of risk factors for BC changed before (mean 65.79, SD 14.63) and after (mean 77.07, SD 16.57) using BrAware at the 0.005 level of significance. Therefore, this implies that it makes sense to conclude that the intervention might be responsible for improving knowledge of BC risk factors, awareness of warning signs, and confidence in breast self-examination (BSE) among the participants (Table 2).

**Table 2:** Breast cancer awareness (*n* = 41).

**Variable**	**Knowledge**		**95% CI for mean different**	***r***	***t* statistic (df)**	*p*-value*
	**Pre (mean, SD)**	**Post (mean, SD)**				
BC warning signs	70.62 (11.74)	79.83 (10.15)	−13.79, −4.64	0.130	−4.07 (40)	< 0.001
Knowledge of BSE	73.66 (18.94)	83.41 (10.63)	−16.69, −2.84	−0.026	−2.84 (40)	0.007
Knowledge of risk factors for BC	65.79 (14.63)	77.07 (16.57)	−18.95, −3.61	−0.209	−2.97 (40)	0.005

^*^Paired t-test.

## Discussion

The pre and post-tests show an improvement in knowledge of BC among the women population after using the BrAware app. As Nasution et al. (6) mentioned, it is best to approach their knowledge and awareness to change human behavior and promote early BC detection in the community (6). Therefore, the BrAware app can be considered feasible in a real clinical context to promote behavioral changes in the lifestyles of women in performing BSE and screening. The plausible explanation was the regular practice of correct skills while using the app may increase the users' memorisation (13, 14). Most of the participants who had secondary-level education would easily understand simple point-form sentences in the app. This study highlights the importance of mobile apps based on how people's health and well-being can be improved *via* monitoring (15). This result matches those observed in a study in Iran that implemented the smartphone app, which improved BSE practice and even reported abnormal findings among participants by mass palpation or visually inspected nipple retraction (16). According to the Health Belief Model (HBM), an individual's opinion that she is vulnerable to BC, the severity of BC, and the benefits, as well as a barrier to preventative action such as doing the BSE and screening at the hospital, all influence health-seeking behavior toward BC prevention (6). In addition, to our knowledge, no BC awareness support apps are targeting Malaysian women.

A combination of dual language strategies of knowledge dissemination will help facilitate the knowledge transfer to the user as most apps do not provide educational content in the local language. The Malay language is used widely in ASEAN as the official language of Indonesia, Brunei, Singapore and Malaysia, and a lingua franca spoken by communities in southern Thailand, southern Philippines, and parts of Myanmar and Cambodia (17). Therefore, the BrAware app can be a user-friendly tool for the Malaysian community and ASEAN countries on BC. This app provides credible information on breast anatomy, BC, risk factors of BC, treatment modalities, BSE, screening examination, doctor examination, survival rate, finding support group, hotline number, screening reminders and myths and facts based on Malaysian cultural beliefs. The credibility of the BrAware app content is supported by the source of reference info on each page.

Meanwhile, another study promotes the app's credibility by enabling direct communication with the therapist (16). Furthermore, the reminder feature notifications as cues in the app improve user engagement and BSE routine in a time duration set according to the user's menstrual cycle (18). Many studies have supported mobile apps in improving the user's or patient's knowledge and awareness of the diseases (6). A study in China suggested that using a mobile app could improve the user's experience, especially on the accessibility to health information, leading to positive health outcomes (19). Another time-intervention effect study involving 50 years and older group population in Kedah, Malaysia, showed a significantly improved overall knowledge among participants in the intervention group compared with the control group about colorectal cancer (20). Therefore, this study shows that the BrAware app can be fully used to deliver health education and promotion to intended users and enhance the effect of education to change people's behavior.

In the present advancement in communication and digital technology, it can be concluded that the BrAware app is a way forward for health promotion and education, particularly in preventing and early detection of BC. However, there are potential limitations of this study that should be noted. First, the results may not be generalisable to all women in Malaysia as only one state was selected. Therefore, the participants need to be expanded to urbanized women in Malaysia. Also, since this study evaluated the outcomes after 2 months, it is not certain what the knowledge of BC awareness retention is and how long it will be retained. Therefore, we will need to evaluate the app's effectiveness over the long term in the future.

## Data availability statement

The original contributions presented in the study are included in the article/supplementary material, further inquiries can be directed to the corresponding author.

## Ethics statement

The studies involving human participants were reviewed and approved by Human Research Ethics Committee (HREC) of Universiti Sains Malaysia (USM/JEPeM/18080380). The patients/participants provided their written informed consent to participate in this study. Written informed consent was obtained from the individual(s) for the publication of any potentially identifiable images or data included in this article.

## Author contributions

AY, YP, IA, and SL contributed to the conception and design of the study. AY and AN implemented the methods and execution of the study. AY performed the statistical analysis. AY, AN, and SL wrote the manuscript. All authors contributed to manuscript revision, read, and approved the submitted version.

## Funding

This work was supported by the USM short-term grant (STG/304.PPSK.6315141).

## Conflict of interest

The authors declare that the research was conducted in the absence of any commercial or financial relationships that could be construed as a potential conflict of interest.

## Publisher's note

All claims expressed in this article are solely those of the authors and do not necessarily represent those of their affiliated organizations, or those of the publisher, the editors and the reviewers. Any product that may be evaluated in this article, or claim that may be made by its manufacturer, is not guaranteed or endorsed by the publisher.
